# What makes AI ‘intelligent’ and ‘caring’? Exploring affect and relationality across three sites of intelligence and care

**DOI:** 10.1016/j.socscimed.2021.113874

**Published:** 2021-05

**Authors:** Giulia De Togni, Sonja Erikainen, Sarah Chan, Sarah Cunningham-Burley

**Affiliations:** Centre for Biomedicine, Self and Society (CBSS) - USHER Institute - University of Edinburgh Medical School: Molecular, Genetic and Population Health Sciences, United Kingdom

**Keywords:** Artificial intelligence, Health and care, Relationality, Affect, Social computing, Caring machines, Robot ethics

## Abstract

This paper scrutinises how AI and robotic technologies are transforming the relationships between people and machines in new affective, embodied and relational ways. Through investigating what it means to exist as human ‘in relation’ to AI across health and care contexts, we aim to make three main contributions. (1) We start by highlighting the complexities of philosophical issues surrounding the concepts of “artificial intelligence” and “ethical machines.” (2) We outline some potential challenges and opportunities that the creation of such technologies may bring in the health and care settings. We focus on AI applications that interface with health and care via examples where AI is explicitly designed as an ‘augmenting’ technology that can overcome human bodily and cognitive as well as socio-economic constraints. We focus on three dimensions of ‘intelligence’ - physical, interpretive, and emotional - using the examples of robotic surgery, digital pathology, and robot caregivers, respectively. Through investigating these areas, we interrogate the social context and implications of human-technology interaction in the interrelational sphere of care practice. (3) We argue, in conclusion, that there is a need for an interdisciplinary mode of theorising ‘intelligence’ as relational and affective in ways that can accommodate the fragmentation of both conceptual and material boundaries between human and AI, and human and machine. Our aim in investigating these sociological, philosophical and ethical questions is primarily to explore the relationship between affect, relationality and ‘intelligence,’ the intersection and integration of ‘human’ and ‘artificial’ intelligence, through an examination of how AI is used across different dimensions of intelligence. This allows us to scrutinise how ‘intelligence’ is ultimately conveyed, understood and (technologically or algorithmically) configured in practice through emerging relationships that go beyond the conceptual divisions between humans and machines, and humans vis-à-vis artificial intelligence-based technologies.

## Highlights

• Challenges and opportunities of artificial intelligence in health and care.
• Conceptual issues surrounding AI, ethical machines and the human-machine boundary.
• Dimensions of AI in health: robotic surgery, digital pathology, robot caregivers.
• Interdisciplinary mode of theorising ‘intelligence’ as relational and affective.
• Intersection and integration of ‘human’ and ‘artificial’ intelligence.

## 1 Introduction

AI has the potential to be applied in almost all areas of health and care (**Ramesh et al. 2004**). Recent innovations in this field include AI-assisted robotic surgery, pattern recognition in diagnostic pathology, and assistive care robotics. Many of these applications require complex interface technologies and machine learning that can support relational human-AI interaction. While the interpretative, affective, and physical capacities these technologies exhibit can be described as ‘artificial intelligence,’ the conceptual boundaries of ‘intelligence’ as well as ‘artificiality’ remain disputed and open. Over the past decades, ‘intelligence’ has acquired an umbrella character encompassing multiple dimensions beyond abstract logical reasoning, including emotional and physical intelligence (**Sternberg 2000**). Concurrently, philosophical conceptualisations around the extent and nature of ‘human’ vs ‘artificial’ cognitive capacities remain plural and contested (**Robinson 2014**). This contested conceptual landscape is evident in a recent report on AI that defines ‘intelligence’ broadly as “problem-solving,” and “artificially intelligent systems” as taking “the best possible action in a given situation,” leaving unresolved what intelligent “problem-solving” or “best possible action” might actually imply (**Fenech et al., 2018**: 9). What is clear, however, is that ‘emotional’ and ‘relational’ parameters of ‘intelligence’ must comprise a key component of artificial intelligence systems (**Scheutz 2014**).

In this paper, we draw on interdisciplinary conceptual analysis combining our expertise in medical sociology and anthropology, bioethics and philosophy to propose an interdisciplinary mode of theorising ‘intelligence’ in health and care contexts as relational and affective, as a way of making sense of the fragmentation of both conceptual and material boundaries between humans and ‘intelligent,’ ‘caring’ machines. We are particularly interested in exploring the ways in which AI and robotic technologies are generative of new forms of affective relationality including in the ways people give or receive health and social care in such technologically mediated contexts. In accord with **Röttger-Rössler and Slaby** (**2018**: 2), we understand affect not as processes “within” a person, but as social-relational dynamics unfolding in situated practices and social interaction. Our conceptual analysis draws on relevant scientific, social scientific and humanities literature, policy reports, and commissioning guidance on the development of AI and robotic technologies in health and care. Particular attention was given to tracing the anticipatory discourse across these texts in order to investigate the implications of the promise of intelligent, ethical, and affective machines across different health and care applications where different dimensions of intelligence (physical, interpretive and emotional) are embedded. We explore what this might mean for the web of human and non-human relationships in these settings.

Based on this analysis, we first address conceptual complexities surrounding the concept of intelligence and its relationship to the human/machine boundary, ethics, affect and relationality. Second, we consider three different dimensions of intelligence, where the human and machine exist in a co-constitutive relationship; namely, physical, interpretive, and emotional, via the examples of robotic surgery, digital pathology, and care robotics. In so doing, we interrogate how current and anticipated developments in AI and robotics can enable us to think beyond reductive notions of intelligence and human-machine boundaries. Through an exploration of these different dimensions of intelligence instantiated in different health and care contexts, we go on to argue that AI technologies and robotics not only re-materialise the boundaries of the human and the machine in affective and relational ways that challenge old distinctions and binaries between the artificial and natural, rational and emotional, and human and non-human, but they do so by augmenting and, indeed, changing human capabilities.

We conclude that there is a time-critical need for an interdisciplinary approach to theorising intelligence, whether artificial or otherwise, as both relational and affective. Ultimately, we aim to contribute to such theorisation, through this contribution to understanding the relational and affective dimensions in human as well as machine intelligence across different sites of health and care. Indeed, as we show, AI and robotics - through surgery, diagnostics, and care - are already influencing humans’ relationality, affects, and embodiment. Additionally, we hope that our research will inform policy and help those involved in AI, health and care to deal with some of the challenges unpacked in this paper.

## 2 Conceptualising (artificial) intelligence

### 2.1 Human VS machine

AI has been described as “the science and engineering of making intelligent machines” **McCarthy** (**2007**: 2) or “the activity devoted to making machines intelligent” (**Nilsson 2009**: 13). Such definitions raise questions around what ‘intelligence’ itself entails. The word implies a philosophical and cultural fascination with intelligence as a characteristic capacity that seemingly confers on humans a special place among other life forms. The notion of ‘artificial intelligence’ suggests that intelligence can be simulated through technological means, and yet that such simulated intelligence remains different from the ‘natural’ or, perhaps, ‘real’ intelligence exhibited by humans. The meaning of intelligence and how it should be defined and measured in practice remains significantly contested. Although **Fenech et al. (2018)** define intelligence in terms of problem-solving, multiple alternative definitions exist that range from logical or abstract to practical reasoning: for instance, the capacity to learn from experience or to adapt to new situations to achieve one's goals in the confines of one's sociocultural context (**Legg and Hutter 2007**). Correspondingly, definitions of artificial intelligence are plural in ways that reflect the underlying conceptual ambiguity around intelligence in general. Such conceptual ambiguity within and between artificial and human intelligence leads us, not to seek a stable definition, but to examine the interrelatedness of human and non-human dimensions of intelligence, their permeable boundaries and the possibilities of ‘posthuman’ (artificial) intelligence.

Given the complexity of human intelligence in all its facets, theories of intelligence have moved away from models based on abstract reasoning and logic (alone), towards more complex and nuanced frameworks that encompass multiple attributes and dimensions of (human) abilities. For instance, **Gardner**'s (**1993**) multiple intelligences theory incorporates inter- and intrapersonal bodily kinaesthetic, spatial, linguistic and musical dimensions in addition to the logical-mathematical dimension into a plural framework of intelligence. Increasing amounts of research have been devoted to understanding the forms of intelligence required for social interactions and relationships, and the emotional intelligence concept especially has been popularised in both public and academic contexts (**Monnier 2015**). Yet, as with the theory of intelligence in general, there is no commonly agreed definition over the meaning and scope of these multiple forms of intelligence. While computers today are superior to humans in chess and exhibit far greater intelligence in terms of computation power and ability to process large quantities of information at high speed, mapping more nuanced notions of intelligence like emotional or inter-personal aptitudes into artificial systems remains a notable challenge.

Nowadays, the focus of posthuman thought is on shifting away from the humanistic paradigm, deconstructing the notion of human uniqueness including in terms of emotional capabilities, and leading towards the future creation of machines that feel, and initiate feelings in return (**Braidotti 2019**). Posthuman prospects envision possibilities where emotional capacity in AI technologies and robotics no longer separates humans and machines. Indeed, transformations of affective embodiment and material experiences in AI technologies and robotics are already shaping the present and future of health and care, as we show in subsequent sections.

### 2.2 ‘Caring’ AI

The conceptual ambiguities around (human vs machine) intelligence bear directly upon questions around the integration of social and affective dimensions into ‘artificial’ systems, which has been a significant part of innovation in AI (**Wilson, 2009**). Social computing focuses on the use of computational devices to facilitate social interactions among users (e.g. social media), whereas, affective computing draws on an interdisciplinary interest in the non-verbal, often trans-subjective and, at times, non-conscious dimensions of embodied experiences and forms of knowing including feeling, sensation, attention, and listening (**Blackman and Venn 2010**). Pioneered in the early ‘90s by Rosalind Picard, the turn to affect in AI research now branches into wearable computing, big data, psychology, neuroscience, and modelling in order to advance the knowledge, understanding, and development of systems for sensing, recognising, categorising, and reacting to human emotion (**Lee and Norman 2016****)**.

**Picard** (**1997**: 250) and her team at MIT Media Lab have called for “a change in computing” and “rethinking how computers function,” recognising that affect is “integral to human intelligent functioning” and “not a separate process from cognition” but rather “inextricably intertwined with it.” The intersection of AI and affective computing has particular relevance for health and care innovation, where a movement is growing towards more ‘relationship-centered’ and ‘compassionate’ service delivery models (**Beach and Thomas 2006**; **DoH 2009**; **NHS 2012**). These models emphasise the importance of relationships, including the centrality of affect, emotional connection, reciprocity, and their moral as well as therapeutic value in the context of care delivery, where the nature and quality of care relationships influence outcomes (**Dewar and Nolan 2013**).

The notion of affect refers broadly to states of being rather than how they manifest, or are interpreted as emotions like anger, happiness, disgust, or fear (**Hemmings 2005**). Affect is related to but distinct from emotions, and refers to forms of knowledge that entail social interpretation, embodied experiences, and knowings that emerge in relation to other human beings and objects in the world. As such, affect is social-relational (Röttger-Rössler and Slaby 2018) and generated in social contexts through interaction with other people and objects, but in ways also linked with social norms and power relations. AI technologies in health and care are a particularly fruitful example for exploring relational and affective human-technology interfaces. As AI applications are increasingly investigated and implemented into care provision, researchers need to scrutinise critically the implications of human-technology interaction in health and care practice. This is especially the case given that critical analyses of computational algorithms have shown AI applications to be value-laden and configured in ways that privilege some values over others (**Mittelstadt et al. 2016**) an may reflect the power relationships within the health and care settings where they are applied. In the context of care delivery, machine learning introduces increasing complexity by bringing uncertainty to algorithmic decision-making, including about how and what kind of care is provided for different recipients. The intersection of these phenomena opens up a critical space for exploring the empirical, conceptual, and normative foundations and implications of the affective relationality of AI health and care innovations. Furthermore, it raises questions about the relationship between ‘artificiality’ and ‘affectivity,’ and ‘human’ and ‘machine’ intelligence, and about what it might mean relationally to be a care recipient, care provider, or indeed ‘human’ in AI-mediated relationships and spaces in respect to health and care.

### 2.3 ‘Ethical’ machines

AI also raises the possibility of ‘ethical machines,’ that could be conceived as *subjects*, rather than *objects.* These would be ethical agents responsible for their actions, or “autonomous moral agents” (**van Wynsberghe and Robbins 2019**). In this paper, we use the term ‘machines’ in a broad sense to include semi-autonomous and autonomous robots as well as purely algorithmic systems making decisions. Some refer to such machines as “artificial moral agents” (**Allen et al. 2009**) which are supposedly capable of moral decision making (**Anderson et al. 2006**) and thus have an “ethical dimension” (**Crnkovic et al. 2012**). For **Moor (2006)**, a machine counts as a “full ethical agent” if it is comparable to human moral decision makers. **Cave et al.** (**2018**:2) prefer referring to ‘ethically aligned machines,’ machines that “function in a way which is ethically desirable, or at least ethically acceptable” and that are capable of “ethical reasoning.” Yet a wide conceptual gulf remains between machines that simply do what is ethical, and the possibility of genuine artificial moral agency.

In the discussion about reasoning machines, it is commonly agreed that a standard account of agency requires the capacity for intentional actions. A robot that is *incapable* of acting unethically, that merely executes an algorithm, without the capacity for choice and intention, cannot be said to be an agent in this sense. Yet, if we take a relational approach to understanding (artificial) intelligence, the location of agency itself changes to something achieved through interaction. A robot that is programmed to follow ethical rules can very easily be modified, through human intervention, to follow unethical rules (**Vanderelst and Winfield 2018**). As with intelligence, the very concept of what is ethical, as well as what it is to act ethically, is contested and often context-dependent, again demanding a relational, and as we go on to argue, an inter/transdisciplinary approach.

The kind of ‘strong’ or ‘super’ AIs that might have the capacity for genuine moral agency remain the purview of science fiction (**Bostrom 2003**). AI technologies in their present state might be more accurately conceptualised as a form of ‘human augmentation,’ increasing human productivity, capability, or adding to the human body or mind in some way, including through augmented sensing, cognition, and action (**Raisamo et al. 2019**). Even as such, they will nonetheless function as ‘ethical machines’ in the relational sense. What is of interest, therefore, is not the ‘ethical machine’ in isolation and whether AIs may have the capacity for ethical reasoning in themselves, but the ‘ethical human-machine system’. AI technologies, as they augment human capacities, also embed and respond to the interests and values of stakeholders in a given socio-political context, together with the humans that build, interact with, care for and are cared for by them.

AI and robotic applications in the health and care sectors raise a number of ethical challenges including: potential privacy issues; concerns over liberty and dignity of those receiving care (**Sparrow and Sparrow 2006**; **Sharkey and Sharkey 2011**; **Sharkey 2014**); the consequences, both for caregivers and recipients, of replacing human interaction and human caregiving labour with machine substitutes (**Decker 2008**; **Stanford University 2016**); how to deal with error and responsibility concerning machine decisions and actions (**Matthias 2004**; **Sparrow 2007**); how to build trust between machines, patients, and medical professionals; and, especially, their potential to re-enforce social inequalities (**Vallverdú and Casacuberta 2015**). Moreover, as engineers’ views of knowledge affect the way they shape such knowledge in the machines - building a knowledge-based system necessarily involves selection and interpretation (**Forsythe 1993**) - this process opens up possibilities to develop bias, through for instance replicating social stereotypes. One example is the association of femininity with assistive technologies (e.g. Siri, Alexa, Cortana), which sustains ideas of feminine docile labour and replaceable embodiment (**Sutko 2019**). We now go on to reflect on some of these challenges, and how an approach to theorising ethical human-machine systems as relational and affective can offer insights to begin to address them. The three dimensions of intelligence that we discuss below – physical, interpretive, and affective - are a heuristic that we use to explore affect and relationality as AI and robotics enter the world of health and care.

## 3 Three dimensions of ‘intelligence’

1. Physical Intelligence: Robotic Surgery

Robotics integrates information technology and physical embodiment: robots not only inhabit the physical space, but often also physically interact with humans (**Thrun 2004**). The use of increasingly sophisticated algorithms in robotics can facilitate deeper and more complex human-machine relationships with physical dimensions. Robotic surgery, in particular, is a rapidly advancing field primarily aimed at extending human capabilities and overcoming human limitations to improve task performances in surgery (**Camarillo et al. 2004**). Different levels of robotic involvement may be classified broadly into three degrees of autonomy: the passive role, where the robot's involvement is limited in scope; the restricted-active role, where the robot takes more invasive tasks, but is still not actively involved in the essential portions of the procedure; and the active role, where the robot is “intimately involved in the procedure and carries high responsibility and risk” (ibid.: 4S). Even robots that perform an active role, however, have limited capacity for autonomous action. Rather than replacing human surgeons, these machines generally require significant human supervision and interaction, which means that robotic surgery today is more accurately described as ‘robot-assisted’ surgery.

An example of an active-role surgical robot is the daVinci® Surgical System, a tele-operated system where a human surgeon controls the motion of the robot via a remote console (**Intuitive** **Surgical 2019**). The surgeon is connected to the robot via a highly magnified, high definition visual display and control handles that establish a haptic interface between the surgeon and the robot. The control algorithms of the system translate the surgeon's hand movements into the same movements by the robot's surgical instruments, at the same time as the surgeon can receive sensation of pressure and force from the instruments through the haptic feedback. While the daVinci® robot has a low level of autonomy, the robot is engaged in direct and sustained physical contact with the patient's tissues throughout the procedure, giving it an active role within the surgical process (**Camarillo et al. 2004**). The sustained physical contact it has with the patient also means that human-robot relationality is established between the robot and the patient, and between the human surgeon and the human patient via the robot as a ‘physical mediator.’ Through the haptic interface, the robot's actions are intertwined with both the surgeon's and the patient's body. The robot thus becomes intimately integrated within the surgeon-patient interaction in material ways that re-make the relationship between the surgeon and the patient, as the boundaries of the human body and the machine become entangled. Machines such as the daVinci® system establish a mutuality where the surgeon's accuracy is potentially augmented through the robot, at the same time as the robot relies on the surgeon's embodied knowledge and actions.

Robotic surgery has the potential to facilitate microscale surgical interventions that are extremely challenging or impossible for human surgeons to perform without sophisticated robotic mediation due to the physical size, limited motion control, and dexterity of the human hand (**Camarillo et al. 2004**). Clinical areas where microsurgical robots are anticipated include foetal surgery, ophthalmology, laryngology, and otology, with most existing microsurgery robot prototypes having been developed for these areas. An example of prototype microsurgical robots is a device created by a multidisciplinary research team in Italy, which aims to overcome human limitations in performing laser phono-micro-surgery (a challenging surgical procedure to correct vocal cord abnormalities using a surgical laser beam) (**Mattos et al., 2016**). The device is a robot-assisted laser micromanipulator that eliminates the need for manual control of the laser beam, replacing it with a virtual surgeon-robot interface system; the surgeon can control the laser beam via a touchscreen tablet computer, which provides magnified live videos of the surgical area and assistive functions like real-time feedback on the laser incision depth, by using a stylus pen that functions as a ‘virtual scalpel’ (ibid.). The robot-assisted system can significantly enhance precision, safety, efficacy and quality of microsurgery by augmenting the actuation and sensing capacities of the surgeon.

We can think of these robots in terms of ‘physical intelligence,’ going beyond reasoning-centric definitions of intelligence in conceptualising AI. Robot-assisted surgical systems such as those described above are re-making and re-defining the boundaries of human embodiment and physical capability within the surgical context. As such, robotic surgery can be seen to represent the extension of human physical intelligence via augmented accuracy, dexterity and precision, and extended field of vision provided by machines. The integration of robotic systems within surgical teams also implies a breaking down of clear embodied boundaries and sensations between the human and the robot, as the robot extends the surgeon's body and physical skills ‘artificially’ while relying on the surgeon to function in the first place.

Robot-assisted surgery thus reconstitutes both the physical and embodied scope of the surgeon's intelligence and the physical relations of the surgical encounter. This demands further scrutiny of the physical, relational and affective implications of integrating ‘robot surgeons’ into surgical teams, including in terms of what this means for how the boundaries of the human body are being re-made. The human-machine network extends the body with instruments, modulating the user's movements, knowledge, and affects (**Mühlhoff, 2019**). This leads to a reciprocal co-dependence of users and AI, which is at the core of specific forms of mechanised power and control in human-aided AI. Thus, it becomes important to interrogate the relational dynamics through which users associate with these technologies and, in turn, relate to themselves and others (**Guzman and Lewis 2019**). Such process may impact the surgeons' and patients' embodied experiences, while transforming notions of trust, liability, accountability, relationality, and affects. This then raises questions about the implications of robot surgeons' embodiment for ethics and law, including for instance who is held accountable if something goes wrong.

2. Interpretive Intelligence: Digital Pathology

In pathology, machine learning algorithms are being developed especially for digital image analysis to assist in diagnosis (**Tizhoosh and Pantanowitz 2018**). Over the past two decades, this process has been facilitated by the creation of faster digital networks, cheaper data storage and sharing solutions, and advances in image processing, pattern recognition algorithms, and machine learning (**Salto-Tellez et al. 2018**), along with development and widespread use of ‘digital slides’ (**Al-Janabi et al. 2012**). These technologies have the potential to extend the frontiers of pathology and enable the utilisation and integration of knowledge beyond human limitations (**Niazi et al. 2019**). This is especially so when image analysis can be combined with other kinds of integrated data from different sources such as electronic patient records and ‘-omics’ data (**Holzinger et al., 2017**), as shown by recent studies on the success rates of Google Health breast cancer screening (**McKinney et al. 2020**). However, to learn how to diagnose a disease reliably and effectively, the algorithms still require large sets of high-quality training images annotated by human pathologists and programmers beforehand. As in robotic surgery, there are different levels of algorithmic involvement in pathology, and even machine learning algorithms with the highest levels of autonomy have limited capacity to work independently (**Holzinger et al. 2017**). The application of machine learning algorithms in pathology can most accurately be described as ‘machine-aided’ or ‘computer-assisted’ pathology that is facilitating the emergence of algorithmically ‘augmented pathologists’ (**Tizhoosh and Pantanowitz, 2018**).

The dependence on data collected by human surgeons or clinicians means that these algorithms remain inescapably value-laden, as their operational parameters have been specified by the developers and then configured for use by others with specific desired outcomes in mind that prioritise some interests over others (**Martin 2018**). These algorithms can, at best, ‘objectively’ implement the programmers' and annotators' pre-existing decision patterns (**Tadrious, 2010**). However, the outputs that algorithms produce can never exceed the inputs, which means that the decisions algorithms make are always only as reliable (or ‘neutral’) as the data they are based on. Research shows that flaws in the underlying data, especially gender and racial bias, are inadvertently adopted by algorithms. For instance, **Buolamwini and Gebru (2019)** have raised concerns about the efficiency of automated facial analysis algorithms and databases with respect to phenotypic subgroups. They found that in the U.S. such datasets are overwhelmingly composed of lighter-skinned subjects, with rates of up to 86.2%, and that darker-skinned females are the most misclassified group, with error rates of up to 34.7% (Buolamwini and Gebru 2019: 77). The maximum error rate for lighter-skinned males was instead 0.8%. Studies like these raise urgent ethical questions around how (and whether it is possible) to build fair, transparent, and accountable analysis algorithms. More generally, training data sets may set in a range of biases, not necessarily racial or gendered, which will shape practices in ways that are neither obvious nor transparent.

The above also raises questions about the ways in which racialised affects and especially the unconscious dimensions of affective responses to racial (and other socially significant) differences exhibited by humans become embedded within and reinforced via algorithms. Indeed, as **Al-Saji (2014)** among others has argued, racialisation, while often remaining an unconscious process, takes hold of bodies by means of perception. Individuals project ‘race’ as a property upon a body including by naturalising features like skin colour, facial attributes, etc. as racial features. In the case of image processing, pattern recognition algorithms, and machine learning, questions around fairness, transparency and accountability thus stretch beyond more constrained framings of ‘bias.’ They encompass wider social, cultural and structural forces that shape how humans perceive and categorise difference and response to difference in affective terms. Algorithms will inevitably inherit these perceptions and categorisations and, in doing so, are likely to incite affective responses to them, potentially influencing human responses based on racial biases.

It is also important to investigate how the human interpretation of pathologists increasingly relying on digital images co-analysed by algorithms is substantially changing how disease is interpreted and conceptualised as well as the responses to it. Moreover, there is currently no established way to explain why machine learning algorithms make a particular decision when interpreting digital slide images. This is a manifestation of the wider ‘black box’ problem that pertains to most contexts where these algorithms are employed (**Burrell 2016**). This is especially problematic in medicine where a reliable diagnosis should be transparent and fully comprehensible, while the pathologists and other human practitioners involved need to justify reasons for reaching particular medical decisions (**Tizhoosh and Pantanowitz, 2018**). The result is that it is not straightforward to identify who should be held responsible and accountable for diagnostic errors.

The relationship between human subjectivity, affect, and algorithmic design works both ways: algorithms can also change how humans conceptualise, perceive or (affectively) respond to the world, for example by producing new categories of illness and disease from the data through identifying novel patterns of malignancy or correlations between population sub-groups and types of disease. In the words of **Mittlestandt et al.** (**2016**: 5), “algorithmic activities, like profiling, re-ontologise the world by understanding and conceptualising it in new, unexpected ways, and triggering and motivating actions based on the insights it generates.” This has important implications for medicine in terms of how patients and illnesses are categorised and treated, including when new or emerging categories of illness or disease susceptibility align with pre-existing socially meaningful categories like race or gender. It will also change both pathologists' and patients' experiences of healthcare provision in embodied, relational, and affective terms. Firstly, digital pathology is changing what has conventionally been largely a qualitative assessment of tissue samples by a human pathologist into an increasingly quantitative assessment co-performed by algorithms. This is likely to change the pathologists’ embodied perception of diagnostics, not only due to the digitisation of pathology workflows but also due to the consequent loss of contact with the materiality of the human tissue on the part of the pathologist.

Secondly, the relationships (including of trust) that patients have with human physicians will be conditioned by algorithms as central actors in the making of their diagnoses, while the shift from traditional pathology and patient care to digital pathology will require technological solutions that pull information from a wide range of disparate medical databases. The convergence of advanced imaging, automation, and powerful analytics like natural language processing and machine learning, are bringing together the tools needed for scientists and clinicians to make medical breakthroughs at an unprecedented pace. It is thus crucial to analyse how these technologies are transforming practices of health and care and to critically interrogate the meaning and nature of ‘fair,’ ‘inclusive,’ ‘transparent’ and ‘accountable’ analysis algorithms. Thus, concerns that have been raised around fairness, transparency, bias and accountability with respect to algorithmic medicine must also be read with an understanding of how the shift to digital pathology alters the relational aspects of health and care practices. This includes questions around how algorithms embed and may reinforce socially and culturally conditioned ‘habits of seeing’ and processes of categorisation and interpretation that have the potential to reinforce existing social frameworks of difference and ‘otherness’ as well as to enable medical advances.

3. Emotional Intelligence: Socially Assistive Robots

Robotics in the delivery of care is expected to flourish in the face of shortages of healthcare personnel, ageing populations, and calls for improved quality of care. Developments in AI in combination with assistive physical technologies are currently facilitating the production of Socially Assistive Robots (SARs). These emotionally perceptive or intelligent machines represent a new site of affective relationality in care, designed to interact with humans via a communicative range that includes ‘emotional’ responses (**Ziemke, 2001**). These responses include interaction, communication, companionship, and emotional attachment (**Kolling et al., 2016**). The potential of robot carers has taken on new significance in the context of the COVID-19 pandemic, as a way of mitigating the risks to public and individual health caused by human contact (**Forman et al. 2020**). However, the emergence of SARs also raises new ethical and social as well as technological questions, including about the ways in which emotionality and sociability should be and are being algorithmically configured and how care recipients might perceive and respond to such algorithmic sociability. It is still unclear how the perceived role and value of human relationships might influence the development of SARs, and how these technologies may, in turn, influence the nature of existing relationships between humans. Furthermore, there are likely to be cross-cultural differences in the answers that are given to these questions. For instance, in Japan - which is the leading country in the production of SARs - the emergence of social robotics has already become a site of intense affective and psychological investment, and the attribution of ‘heart/mind’ (*kokoro*) to robots is a common phenomenon (**Katsuno, 2011**). In the Japanese context, **Sugano** (**1997**: 21) argues that to make robots “truly useful for humans, it is ideal to establish a ‘heart-to-heart relationship’ (*kokoro no fureai*), which enables both the human and the robot to understand each other like human beings. In this light, the robot needs its own heart.”

Sugano identifies three types of robot *kokoro*, which pertain to different aspects of the human-robot affective relationship: the robot that affects human *kokoro*; the robot that can understand human *kokoro*; and the robot with its own *kokoro*. The first category of robots capable of affecting human *kokoro* encompasses companion and therapeutic robots, often modelled in the shape of animals, that are designed to affect the mental and emotional states of humans. Examples include the Sony® AIBO, a series of commercial dog companion robots (**Sony 2019**); and the AIST Paro, a soft baby seal robot designed for use in hospitals and nursing homes as a therapeutic tool (**IEEE 2019**). The second category maps onto what **Turkle (2007)** has described as ‘relational artefacts:’ sociable machines equipped with computational systems designed to create a conduit for emotional ‘touch’ with humans by being capable of actively facilitating reciprocal communication. The majority of contemporary research aiming to develop humanoid robots focuses on this type of intelligent and caring machines. Sugano's third category, robots that have their own *kokoro* or their own emotional responses and affective states, remains confined to science fiction as it pertains to ‘super intelligence.’ Nonetheless, emotionally intelligent robots are indubitably present as sociotechnical imaginaries in academic discourse as well as popular culture particularly in Japan.

**Kim and Kim (2012)** assert that ‘culture’ is an important factor to consider when evaluating an individual's emotional attitudes toward robots, because it affects people's beliefs, and behaviours. Drawing on cross-cultural comparative research, Kim and Kim observe that bonding with inanimate objects and cohabiting with a ‘friendly robot’ appear to be deeply embedded particularly in the ‘Japanese culture.’ **Šabanović (2014)**, however, points out that the widespread social acceptability of robots in Japan is the result of technical discourse and practices of robotics researchers, who for decades have adapted their designs to public taste to promote social acceptance of their work. As documented by **Katsuno** (**2011:** 102), while robot builders in Japan “describe the early development of their robots as alter egos, they also notice that […] public expectations of the robot also play a role in the process of ‘raising’ their robots.” Moreover, since the 1950s, popular culture in Japan has contributed to forming the image of a friendly robot through famous iconic animation characters such as ‘Tetsuwan Atom’ and ‘Doraemon.’ **Hornyak (2006)** documents that the Japanese engineers building ‘friendly robots’ had these characters as models in their mind. The specificity of the Japanese socio-historical context demonstrates the need to develop new conceptual tools informed by social analysis to engage with the “contradictions that robot-human relations evoke” in humans (**Shaw-Garlock, 2009****:** 258).

**Wilson (2010)** suggests that our understanding of what constitutes an artificial intelligence and an intelligent machine is deeply inflected by fantasy, performance, and emotion. Experiments have demonstrated that people project life-like attributes onto robots to impute traces of empathy in the machines (**Darling et al. 2015**: 770). In particular, research shows that humans ascribe agency to some robots and treat them as social actors (**Darling 2017**). For instance, **Riek et al. (2009)** carried out an experiment in the U.S. where human participants were shown videos of robots with increasing anthropomorphic attributes being mistreated by humans. The participants were asked to share their feelings about these videos, to say how sorry they felt for each kind of robot and which ones they would like to save in case of an earthquake. Most of the participants chose the humanoid robots over non-anthropomorphic machines, suggesting that anthropomorphism plays and important role in soliciting empathy towards robots.

In fact, human communication and interaction make significant use of complex non-verbal gestures such as facial expressions, hand and body movements, which support the perception of connectedness between the human communicators (**Sidner et al., 2005**). While designing human likeness including non-verbal gesturing into robots may be significant for the creation of trust and ability to bond with robots, **Mori**'s (**1970**) ‘uncanny valley’ hypothesis also suggests that there is a certain threshold beyond which the human-like appearance of a robot may repel rather than attract humans. Eventually, how people emotionally and behaviourally respond to ‘human like’ robots remains unpredictable (**Woods et at. 2004**). As discussed, in Japan socially embedded design and popular culture facilitated the public acceptance of humanised robots. Conversely, ‘Western’ popular culture in particular may have created a more negative image of humanoid robots, through science fiction novels and films like ‘I, Robot’ and ‘The Terminator.’

It is important to consider how robotising social spaces might induce new socio-material ontologies that entangle human and machine sociability and affectivity; and the ways in which scholarly descriptions of the emerging socio-material collectives may ‘forget’ or prioritise some aspects of human personhood at the expense of others (**Jones 2017**). The emergence and development of SARs have the potential to re-define how health and social care is provided, both in relational and affective as well as socio-economic terms. Moreover, building sociable robots could allow to gain a scientific understanding of social intelligence and human sociality (**Breazeal, 2002**: 6). Making socially and emotionally intelligent machines requires a model of social and emotional intelligence that can be configured algorithmically, but the models that are chosen for such configuration will inevitably be shaped by social and cultural notions of what these forms of intelligence look like. SARs are not only designed to trigger human emotions, but the incorporation of such robotic entities into the realm of social life invariably alters the conditions and dynamics of human interaction; this gives rise to a social context where humans co-mingle and live ‘in relation’ with intelligent and caring machines (**Shanyang, 2006**). While it remains uncertain whether computing itself can be ‘affective,’ and what it means for it to be so (**Hollnagel, 2003**), it is crucial to attend to affective dimensions of AI and ethical systems into which they will be integrated, and to scrutinise the consequences of this for reconfigurations of human and machine relationships across all dimensions of (artificial) intelligence and sites of application.

## 4 Conclusion

The potential for both AI and robotics in health and social care is vast, as these technologies are increasingly a part of our healthcare eco-system, actively transforming the relationship between humans and machines in new affective, embodied, and relational ways. In digital pathology, AI is already being successfully used to detect diseases more accurately and in earlier stages, enabling pathologists and other healthcare providers to better diagnose, monitor and treat potentially life-threatening conditions. Combined with AI technologies, robots are also increasingly being used in healthcare, ranging from simple laboratory robots to highly complex surgical robots, which can aid a human surgeon to execute complex operations. In addition to surgery, robots are increasingly used in hospitals and care settings including in rehabilitation, physical therapy and in support of those with long-term conditions such as dementia. Social robots have the potential to revolutionise, in particular but not exclusively, the care for ageing populations helping people to remain independent for longer, reducing the need for hospitalisation and care homes. By exploring these different contexts and the range of intelligence therein, we have begun to show how affective and relational dimensions are being constituted in the development and application of these new modes of health and social care. Understanding what ‘intelligence’ means beyond the conceptual human-machine division allows further understanding of how AI functions as an ‘augmenting’ technology that is moving beyond human bodily, cognitive, and spatial constraints. In this paper, we also began to explore how the ‘intelligent’, ’caring’ and ‘relational’ capacities of AI influence the way in which such ethical systems function, and what the consequences and ethical implications of these relational processes might be.

Highlighting the affective and relational dimensions of AI in health and social care contexts we focused on how three different dimensions of intelligence are conveyed, understood, and configured in and through technological innovation. Our analysis begins to suggest what this means for contemporary health and care practices. In so doing, we have examined how current and anticipated innovations around AI technologies and robotics might be shaping health and social care provision. This simultaneously impacts on the nature of care relationships, and on the nature of the capabilities and ‘intelligences’ that are and can be embodied by human care providers and medical professionals in interaction with these non-human presences. We argue that the current and anticipated near future developments in AI can most accurately be described as ‘human augmentation’ and that such a focus foregrounds the interactional entanglements that do and will shape intelligence, affect and relationships in and across different health and care contexts.

By considering robotic surgery, machine learning algorithms in digital pathology, and care robotics as augmenting technologies that aim to extend human capacities and overcome limitations, we have explored how the boundaries of the human body, interpretive abilities, resource constraints, and affective relational connections are being made and remade through the human-machine interaction. These phenomena demonstrate how the current context of AI innovations in care is less about modelling the multidimensional nature of ‘human intelligence’ into an artificial system, and more about co-constitutive processes through which the boundaries of the human and the machine are being re-configured in relation to each other. Such re-configuration is taking place in ways that are moving beyond reductive conceptual divisions between humans and machines, and human and artificial intelligence. As **Seyfert (2018)** identifies, in a different AI context (high frequency trading), ‘users’ of such technologies become components of the system, developing bonds between humans and machines. Our three exemplars demonstrate that embodied relationship – whether this is the surgeon's augmented dexterity, the pathologist's interpretive reach or the care receiver's social connectedness - and underlies our argument that we need to garner interdisciplinary conceptual apparatus to understand the co-constitutive dynamics of developments in (artificial) intelligence.

Intelligent and caring machines are transforming the mental and physical scope of human health and care providers and recipients, entailing the emergence of new kinds of affective relationships and connections between humans and machines. However, the manifestations and implications of this process currently remain uncertain and in-depth, empirical research needs to be developed alongside more abstract argument about what is or should happen. As non-human AI and robotic actors are increasingly integrated into healthcare teams and relationships, they also give rise to new social and ethical challenges regarding the possible effects that these technologies may have on the quality and efficacy of care, while raising crucial questions of accountability and responsibility over errors and malpractice. Without developing detailed understanding of the fundamental transformations in (artificial) intelligence in practice, where humans and machines form the new eco-system of health and care, we will not be able to ascertain what is lost and gained, by and for whom, or therefore to exercise agency in crafting our future relationships of health and care in transparent and equitable ways.

The use of these technologies also pertains to the ways in which different kinds of capabilities, skills and forms of ‘intelligence’ are being modelled into human-interfacing AI and robotic systems. These timely questions can only be addressed through an interdisciplinary mode of research and scholarship that can accommodate the fragmentation of conceptual as well as material boundaries between humans and technology, and between ‘human’ and ‘artificial’ intelligence, and the consequences of this for practices of health and care. There is thus a need for further development of the conceptual, normative, and ethical tools that are used to understand and evaluate both AI-driven technologies and the changes they are making in the expression and manifestations of the affective and relational aspects of human experience. Through this paper, we hope to have contributed towards this effort by examining how ‘intelligence,’ in its different dimensions, is being manifested and co-constituted through the human-technology interface, in ways that are re-materialising the boundaries of the human and the machine identities in affective, embodied, and relational ways. We enjoin further exploration of AI and robotics in health and social care that centres how intelligence is being understood and created, the affective and relational practices that develop in different contexts, and the implications of this for our health and care practices.

## Credit author statement

1. Dr Giulia De Togni (University of Edinburgh): Conceptualisation; Formal analysis; Investigation; Methodology; Writing Original draft preparation, Reviewing and Editing, and Writing Final draft preparation;

2. Dr Sonja Erikainen (University of Edinburgh): Conceptualisation; Formal analysis; Investigation; Methodology; Writing- Original draft preparation, Reviewing and Editing;

3. Dr Sarah Chan (University of Edinburgh): Conceptualisation; Formal analysis; Investigation; Methodology; Writing-Reviewing and Editing;

4. Dr Sarah Cunningham-Burley (University of Edinburgh): Conceptualisation; Formal analysis; Funding acquisition; Investigation; Methodology; Writing-Reviewing and Editing; and Writing Final draft preparation.

## References

[bib1] Al-Janabi S., Huisman A., Van Diest P. (2012). ‘Digital pathology: current status and future perspectives.’ Histopathology.

[bib2] Al-Saji A., Lee E.S. (2014). ‘A phenomenology of hesitation: interrupting racializing habits of seeing.’. ‘Living Alterities: Phenomenology, Embodiment, and race.’ Albany: State University of New York Press.

[bib3] Allen C., Wallach W. (2009). ‘Moral machines: teaching robots right from wrong.’.

[bib4] Anderson M., Anderson S.L. (2006). ‘Guest Editors’ Introduction: Machine Ethics' *IEEE Intell. Syst.*,.

[bib5] Beach M.C., Thomas I. (2006). ‘The relationship-centered care network.’ in. Relationship-Centered Care: A Constructive Reframing. JGIM.

[bib6] Blackman L., Venn C. (2010). ‘Affect’. Body Soc..

[bib7] Bostrom N. (2003). (2003) ‘ethical issues in advanced artificial intelligence.’ revised version of a paper published in cognitive, emotive and ethical aspects of decision making in humans and artificial intelligence. IASSRC.

[bib8] Braidotti R. (2019). ‘A theoretical framework for the critical posthumanities.’ *theory, culture & society*. November.

[bib9] Breazeal C. (2002). ‘Designing sociable robots.’ cambridge: MIT press.

[bib10] Buolamwini J., Gebru T. (2019). ‘Proceedings of the 1st conference on fairness. Accountability and Transparency’ PMLR.

[bib11] Burrell J. (2016). ‘How the Machine ‘Thinks’: Understanding Opacity in Machine Learning Algorithms.’ Big Data & Society.

[bib12] Camarillo D.B., Krummel T.M., Salisbury J.K. (2004). ‘Robotic technology in surgery: past, present, and future.’ The American Journal of Surgery.

[bib13] Cave S., Nyrup R., Vold K., Weller A. (2018). ‘Motivations and risks of machine ethics’ IEEE.

[bib14] Crnkovic G.D., Çürüklü B. (2012). ‘Robots: ethical by design.’ *ethics inf*. Technol..

[bib15] Darling K. (2017). ‘“Who's johnny?” anthropomorphic framing in human-robot interaction, integration, and policy.” *ROBOT ETHICS 2.0*, eds. P. Lin, G. Bekey, K. Abney, R. Jenkins.

[bib16] Darling K., Palash N., Breazeal C. (2015). (2015) ‘empathic concern and the effect of stories in human-robot interaction.’ *Robot and human interactive communication* (RO-MAN), 2015 24th IEEE international symposium on, IEEE.

[bib17] Decker M. (2008). ‘Caregiving robots and ethical reflection: the perspective of interdisciplinary technology assessment.’. AI Soc..

[bib18] Dewar B., Nolan M. (2013). ‘Caring about caring: developing a model to implement compassionate relationship centred care in an older people care setting.’. Int. J. Nurs. Stud..

[bib19] DoH (2009). ‘The national health service constitution.’ retrieved from. http://www.nhs.uk/choiceintheNHS/Rightsand-pledges/NHSConstitution/Documents/nhs-constitution-interactive-version-march-2012.pdf.

[bib20] Fenech M., Strukelj N., Buston O. (2018). ‘Ethical, social, and political challenges of artificial intelligence in health.’. Future Advocacy and the Wellcome Trust.

[bib21] Forman R., Atun R., McKee M., Mossialos E. (2020). ‘12 Lessons learned from the management of the coronavirus pandemic.’. Health Pol..

[bib22] Forsythe D.E. (1993). ‘Engineering knowledge: the construction of knowledge in artificial intelligence.’. Soc. Stud. Sci..

[bib23] Gardner H. (1993). ‘Frames of mind. Theory of multiple intelligences.’ London: Fontana Press.

[bib24] Guzman A.L., Lewis S.C. (2019). ‘Artificial intelligence and communication: a human–machine communication research agenda.’ *SAGE journals: new media & society*.

[bib25] Hemmings C. (2005). ‘Invoking affect. cultural theory and the ontological turn.’ Cultural studies.

[bib26] Hollnagel Erik (2003). ‘Is affective computing and oxymoron?*’ international Journal of human - computer studies*. 2003.

[bib27] Holzinger A., Malle B., Kieseberg P., Roth P.M., Müller H., Reihs R. (2017). ‘Towards the augmented pathologist: challenges of explainable-AI in digital pathology.’ *cornell university: arXiv preprint* arXiv: 1712.06657.

[bib28] Hornyak T.N. (2006). ‘Loving the Machine.’ Tokyo: Kodansha International.

[bib29] IEEE (2019). ‘PARO therapeutic robot.’ Retrieved from. http://www.parorobots.com.

[bib31] Jones R.A. (2017). ‘What makes a robot ‘social’?*’ Social Studies of Science*. 2017.

[bib32] Katsuno H. (2011). ‘The robot's heart: tinkering with humanity and intimacy in robot-building. Jpn. Stud..

[bib33] Kim M.S., Kim E.J. (2012). ‘Humanoid robots as ‘‘The Cultural Other’’: are we able to love our creations?*’*. AI Soc..

[bib34] Kolling T., Baisch S., Schall A., Selig S., Rühl S., Kim Z., Rossberg H., Klein B., Pantel J., Oswald F., Knopf M. (2016). ‘What Is Emotional about Emotional Robotics?’ in Emotions, Technology, and Health.

[bib35] Lee W., Norman M. (2016). ‘Affective computing as complex systems science.’. Procedia Computer Science.

[bib36] Legg S., Hutter M. (2007). ‘Universal intelligence: a definition of machine intelligence’. *Minds and machines: Journal for artificial intelligence, Philosophy and cognitive sciences*,. Dec.

[bib37] Martin K. (2018). ‘Ethical implications and accountability of algorithms. J. Bus. Ethics.

[bib38] Matthias A. (2004). ‘The responsibility gap: Ascribing responsibility for actions of learning automata.’ Ethics and Information Technology.

[bib39] Mattos L., Caldwell D., Peretti G., Mora F., Guastini L., Congolani R. (2016). ‘Microsurgery robots: addressing the needs of high-precision surgical interventions.’. Swiss medical weekly.

[bib40] McCarthy John (2007). ‘What is artificial intelligence?*’* computer science department. Stanford University.

[bib41] McKinney S.M. (2020). ‘International evaluation of an AI system for breast cancer screening.’. Nature.

[bib42] Mittelstadt B.D., Allo P., Taddeo M., Wachter S., Floridi L. (2016). ‘The ethics of algorithms: mapping the debate.’ *Big data & Society*. November.

[bib43] Monnier M. (2015). ‘Difficulties in defining socio-emotional intelligence, competences and skills – a theoretical analysis and structural suggestion.’. International research in vocational education and training.

[bib44] Moor J.H. (2006). ‘The nature, importance, and difficulty of machine ethics’ *IEEE Intell*. Off. Syst..

[bib45] Mori M. (1970). ‘Bukimi no tani*’* (Lit. “The eerie-feeling valley”). Energy.

[bib46] Mühlhoff R. (2019). ‘Human-aided artificial intelligence: or, how to run large computations in human brains? Toward a media sociology of machine learning.*’* SAGE Journals: new Media & Society.

[bib47] NHS. (2012). ‘Compassion in practice: nursing, midwifery and care staff. Our vision and strategy.’ retrieved from. https://www.england.nhs.uk/wp-content/uploads/2012/12/compassion-in-practice.pdf.

[bib48] Niazi M., Parwani A., Gurcan M. (2019). ‘Digital pathology and artificial intelligence.*’ the lancet oncology*, may. 2019.

[bib49] Nilsson N.J. (2009). ‘The Quest for Artificial Intelligence: A History of Ideas and Achievements.’ Cambridge: Cambridge University Press.

[bib50] Picard R.W. (1997). MIT Press.

[bib51] Raisamo R., Rakkolainen I., Majaranta P., Salminen K., Rantala J. (2019). ‘Human augmentation: Past, present and future.’ International Journal of Human-Computer Studies.

[bib52] Ramesh A.N., Kambhampati C., Monson J.R.T., Drew P.J. (2004). ‘Review: artificial intelligence in medicine.’. Ann. R. Coll. Surg. Engl..

[bib53] Riek L.D., Rabinowitch T., Chakrabarti B., Robinson P. (2009). ‘How anthropomorphism affects empathy toward robots.’. Proceedings of the 4th ACM/IEEE International Conference on Human-Robot Interaction.

[bib54] Robinson W., Frankish K., Ramsey W. (2014). ‘Philosophical Challenges.’. The Cambridge Handbook of Artificial Intelligence.

[bib55] Röttger-Rössler B., Slaby J. (2018). Families, places and technologies.’ london and New York. ‘Affect in Relation.

[bib56] Šabanović S. (2014). ‘Inventing Japan's 'robotics culture': the repeated assemble of science, technology, and culture in social robotics.*’* in. Soc. Stud. Sci..

[bib57] Salto-Tellez M., Maxwell P., Hamilton P.W. (2018). ‘Artificial intelligence - the third revolution in pathology.’. Histopathology.

[bib58] Scheutz M., Frankish K., Ramsey W. (2014). ‘Artificial Emotions and Machine Consciousness.’. ‘The Cambridge Handbook of Artificial Intelligence’.

[bib59] Seyfert R., Röttger-Rössler B., Slaby J. (2018). ‘Automation and affect: a study of algorithmic trading.’. op.cit.

[bib60] Shanyang Zhao (2006). ‘Humanoid social robots as a medium of communication.*’* SAGE journals: new media & society. Vol8(3).

[bib61] Sharkey A. (2014). ‘Robots and human dignity: a consideration of the effects of robot care on the dignity of older people.’. Ethics Inf. Technol..

[bib62] Sharkey N., Sharkey A., Lin Patrick, Abney Keith, Bekey George (2011).

[bib63] Shaw-Garlock G. (2009). ‘Looking forward to sociable robots.’. International Journal of Social Robotics.

[bib64] Sidner C., Lee C., Kidd C., Lesh N., Rich C. (2005). ‘Explorations in engagement for humans and robots.’. Artif. Intell..

[bib65] Sony (2019). ‘Aibo.’ retrieved from. https://us.aibo.com.

[bib66] Sparrow R. (2007). ‘Killer robots. J. Appl. Philos..

[bib67] Sparrow R., Sparrow L. (2006). ‘In the hands of machines? The future of aged care.’. Minds Mach..

[bib68] Stanford University (2016). ‘One hundred year study on artificial intelligence (AI 100): artificial intelligence and life in 2030.’ report retrieved from. https://ai100.stanford.edu/sites/g/files/sbiybj9861/f/ai_100_report_0906fnlc_single.pd.

[bib69] Sternberg R. (2000). ‘Handbook of Intelligence.’.

[bib70] Sugano S. (1997). ‘Robotto to ningen no kokoro no intafēsu*’* [“Formation of Mind in Robots for Human-Interface”]. Society of Biomechanisms Japan 21.

[bib71] Surgical Intuitive (2019). ‘Da vinci by intuitive.’ retrieved from. https://www.intuitive.com/en-us/products-and-services/da-vinci.

[bib72] Sutko D.M. (2019). ‘Theorizing femininity in artificial intelligence: a framework for undoing technology's gender troubles. ’ Routledge Cultural Studies.

[bib73] Tadrious P.J. (2010). ‘On the concept of objectivity in digital image analysis in pathology.’. Pathology.

[bib74] Thrun S. (2004). ‘Toward a framework of human-robot interaction.’. Hum. Comput. Interact..

[bib75] Tizhoosh H.R., Pantanowitz L. (2018). ‘Artificial Intelligence and Digital Pathology: Challenges and Opportunities.’ Journal of Pathology Informatics.

[bib76] Turkle S., S (2007). Introduction: the things that matter,’ in Turkle. ‘ *Evocative Objects: Things We Think with*. Cambridge MA.

[bib77] Vallverdú J., Casacuberta D., van Rysewyk S., Pontier M. (2015). ‘Ethical and technical aspects of emotions to create empathy in medical machines.’. ‘Machine Medical Ethics. Intelligent Systems, Control and Automation: Science and Engineering.’.

[bib78] Vanderelst D., Winfield A. (2018). ‘The Dark Side of Ethical Robots,’ in *Proceedings of the 2018 AAAI/ACM Conference on AI, Ethics, and Society*.

[bib79] Wilson E.A. (2009). “‘Would I had him with me always’ affects of longing in early artificial intelligence.”. The History Science Society.

[bib80] Wilson E. (2010). A. ‘Affect and Artificial Intelligence.’ Seattle/London.

[bib81] Woods S., Dautenhahn K., Schulz J. (2004). ‘The design space of robots: investigating children's views.’. Proceedings of the 13th IEEE International Workshop on Robot and Human Interactive Communication, RO-MAN.

[bib82] van Wynsberghe A., Robbins S. (2019). ‘Critiquing the reasons for making artificial moral agents,’. Sci. Eng. Ethics.

[bib83] Ziemke T. (2001). ‘Are Robots Embodied?*’ Proceedings from Int. Workshop on Epigenetic Robotics*.

